# Temporal Order of Sexual Offending Is Risk-Relevant for Individuals With Child Sexual Exploitation Materials Offences

**DOI:** 10.5964/sotrap.7229

**Published:** 2022-12-13

**Authors:** Kelly M. Babchishin, Seung C. Lee, Angela W. Eke, Michael C. Seto

**Affiliations:** 1 Department of Psychology, Carleton University, Ottawa, Ontario, Canada; 2 University of Ottawa’s Institute of Mental Health Research, The Royal Ottawa Health Care Group, Ottawa, Ontario, Canada; 3 Public Safety Canada, Ottawa, Ontario, Canada; 4 Criminal Behaviour Analysis Section, Ontario Provincial Police, Orillia, Ontario, Canada; University Medical Center Mainz, Mainz, Germany

**Keywords:** child pornography, child sexual exploitation material, recidivism, risk factor, escalation

## Abstract

The current study examined the extent to which the temporal order of sexual offending may be risk-relevant for men with Child Sexual Exploitation Material (CSEM; also referred to as child pornography) offences. We categorized 85 men who had committed two distinct sexual offences (CSEM or contact sexual offence) into three groups: (1) 47% (n = 40) followed a stable pattern, that is, men with CSEM offences who then committed a new CSEM offence; (2) 41% (n = 35) followed a de-escalation pattern, that is, men with contact sexual offences who then committed a CSEM offence; (3) and 12% (n = 10) followed an escalation pattern, that is, men with CSEM offences who then committed a contact sexual offence. Compared to the other groups, the stable group had more sexual interest in children, the de-escalation group had a younger age at first police involvement and more prior offending, and the escalation group had more substance use problems. We then examined recidivism (any new offence after the second sexual offence) and found that the escalation group had the highest 5-year and 7-year reoffending rates (start of follow-up: opportunity after the second sexual offence) for any crime, any non-sexual violence, any violence (including contact sexual offences), and any contact sexual recidivism. The de-escalation and stable groups had the highest CSEM recidivism rates. The current study suggests that ordering of offending within men adjudicated for CSEM offences is risk-relevant and that those who fit the escalation pattern may be at higher risk to reoffend.

## Abstract (plain language summary)

**Background:**

Individuals with Child Sexual Exploitation Materials (CSEM; also referred to legally as child pornography in Canada and the United States) offences now represent a large proportion of caseloads of sexual offences. Among those with both contact sexual offences and CSEM offences, it is not yet known whether the order of sexual offences (i.e., contact then CSEM or CSEM then contact) is related to risk of reoffending.

**Why was this study done?:**

The order of the sexual offences in mixed CSEM offending ‒ whether a CSEM offence was committed before or after a contact sexual offence – and its association with risk factors and future reoffending has not yet been examined.

**What did the researchers do and find?:**

From a sample of 387 men adjudicated for CSEM offences, we categorized the 85 men who had committed two distinct sexual offences (CSEM or contact sexual offence) into three groups: (1) 47% (n = 40) followed a stable pattern, that is, men with CSEM offences who then committed a new CSEM offence; (2) 41% (n = 35) followed a de-escalation pattern, that is, men with contact sexual offences who then committed a CSEM offence; (3) and 12% (n = 10) followed an escalation pattern, that is, men with CSEM offences who then committed a contact sexual offence. We found that men who fit the escalation pattern (CSEM then contact sexual offence) scored higher on risk factors and had higher recidivism rates.

**What do these findings mean?:**

The management and treatment of individuals with CSEM offending should be sensitive to their sexual offending patterns.

## Highlights

- Ordering of offending within men adjudicated for Child Sexual Exploitation Materials (CSEM) offences is risk-relevant.
- Men who fit the escalation pattern (CSEM then contact sexual offence) may be at a relatively higher risk to reoffend.
- A significant proportion (8%) of men who fit the stable pattern (CSEM then CSEM) commit a contact sexual offence after 5 and 7 years.
- The management and treatment of individuals with CSEM offending should be sensitive to their sexual offending patterns.

Individuals with Child Sexual Exploitation Materials (CSEM; also referred to legally as child pornography in Canada and the United States) offences represent a large proportion of caseloads of sexual offences seen by police, corrections, community supervision officers, and treatment providers. In Canada, the rate of CSEM reported to police increased by 288% from 2010 to 2017 (Department of Justice Canada, 2019), echoing trends of year after year increases in CSEM cases in other countries (UK: McManus & Almond, 2014; US: Adams & Flynn, 2017). The COVID-19 pandemic has further accelerated CSEM offending (Europol, 2020; Wright, 2020), as well as the use of online legal pornography (Wright, 2020). Research direction in terms of management and sentencing are thus essential to support evidence-based practice.

Individuals with CSEM offences have heterogeneous offending patterns. Approximately 1 in 8 have a prior contact sexual offence (i.e., mixed offence type, 15.4% in Elliott et al., 2019; 12.2% in Seto et al., 2011), and 1 in 10 men with CSEM offences have at least one prior nonsexual offence (11.8%; Babchishin et al., 2011). Self-reported rates of crimes are higher than official rates among men with CSEM offences, with approximately half of men with CSEM offences reporting contact sexual offences (Seto et al., 2011). Of individuals with both CSEM and contact sexual offences, we do not know yet the extent to which the temporal order of sexual offences is risk-relevant. That is, mixed offending individuals could escalate (CSEM to contact) or de-escalate (contact to CSEM). The first group (escalation) is of particular public interest because many policies and laws ‒ such as lengthy sentences for CSEM offences in the US (Hamilton, 2011) are based on the assumption that CSEM is a gateway offence for contact sexual offending (Steiker, 2013). The gateway hypothesis presupposes that participating in lower degree antisocial or problematic behaviours increases one’s chance of engaging in higher degree antisocial or problematic behaviours in the future.

Support for the gateway hypothesis – that less severe problematic behaviour can lead to more severe problematic behaviour – has been found for substance use, including tobacco use to illicit drugs (Prescott, 1976), legal and medically prescribed drugs to illegal drugs, as well as from alcohol to illicit drugs (Kandel & Logan, 1984; Yamaguchi & Kandel, 1984). In addition, despite many youths desisting in aggression, an important proportion escalate in severity. Specifically, some boys begin with minor aggression (e.g., bullying), minor delinquency (e.g., shoplifting), or authority problems (e.g., truancy), move to minor violence (e.g., physical fighting) or property damage (e.g., vandalism), and eventually commit serious violence (e.g., sexual offences) or serious delinquency (e.g., burglary; for review, see Loeber & Hay, 1997).

The offending patterns of men with CSEM offences may be meaningful, as individuals with mixed CSEM offending (i.e., individuals who have committed both CSEM and contact sexual offences) have been found to differ from individuals with CSEM-exclusive offending (i.e., those with CSEM offences but no contact sexual offences) and individuals with exclusively contact sexual offences (i.e., those without CSEM offences) on motivational, facilitative, and situational factors for sexual offending (Babchishin et al., 2015; Elliott et al., 2019; Henshaw et al., 2018). Motivational factors largely relate to having an atypical sexual interest for the sexual behaviour (e.g., pedophilia and accessing CSEM or having sexual contact with children), facilitative factors can increase the likelihood of acting on those motivations (e.g., antisocial personality traits, cognitions supportive of sexual offending), and situational factors are those that can provide an opportunity to offend (e.g., access to child victims). A meta-analysis found that men with CSEM-exclusive offending scored lower than men with contact-exclusive offending or men with mixed CSEM offending on measures of antisocial tendencies, hostility, criminal history, substance misuse, and unemployment (i.e., facilitative factors; Babchishin et al., 2015). Mixed offending men were more likely to be pedophilic ‒ a motivational factor ‒ than men with exclusive CSEM offences (Babchishin et al., 2015), who in turn were more likely to be pedophilic than men with exclusively contact sexual offences against children (Babchishin et al., 2015; see also Seto et al., 2006; Seto et al., 2017).

Recidivism studies of men with exclusively CSEM offences suggest that few will be adjudicated for contact sexual offences in the future, whereas mixed offending men are more likely to reoffend with contact sexual offences. For example, a UK study with an average follow-up of 13 years found that 3% of the 584 men with CSEM-exclusive offending had a new conviction for a contact sexual offence compared to 9% of the 106 mixed offending men (Elliott et al., 2019). A large cohort study of 4,658 men with CSEM offending found that, after 5 years of opportunity, 0.3% of men with CSEM-exclusive sexual offences committed a new contact sexual offence compared to 6% of men with mixed CSEM offending (Goller et al., 2010; Graf & Dittmann, 2011). To put this in perspective, the recidivism rates of men with CSEM-exclusive offending are similar to the sexual recidivism rates of men adjudicated for non-sexual offences, which is approximately 1 to 2% after 5 years (*N* = 543,024; Kahn et al., 2017). In contrast, the 5-year contact sexual recidivism rate of men with mixed CSEM offending (6% to 8%; Eke et al., 2019; Goller et al., 2010) is comparable to the rate of men with typical contact sexual offending; that is, men who have an average risk score on the Static-99R (score of 1 to 3; Static-99R [Helmus et al., 2012], the most commonly used actuarial risk assessment for men convicted of sexual offences; Kelley et al., 2020; Neal & Grisso, 2014) who have expected 5-year sexual recidivism rates from 4% to 8% (Hanson, Babchishin, et al., 2017).

Following the Risk, Need, and Responsivity (RNR) principles of correctional rehabilitation (Andrews & Bonta, 2010; Bonta & Andrews, 2016), individuals with a lower risk to reoffend should receive little to no correctional treatment (see also Hanson, Bourgon, et al., 2017 for suggested dosage thresholds). In line with principles of effective correctional rehabilitation, the majority of treatment programs designed for CSEM offending are similar to contact sexual offending programs in treatment targets but have lower treatment dosage (for review, see Paquette et al., 2020). Treatment evaluations for individuals with CSEM offending that examined reoffending, however, have found no difference in offending between treatment and control groups (Beier et al., 2015; Wild et al., 2020; see also Table A.8 in Mews et al., 2017). One potential explanation is that the programs that target those with any CSEM offences overlook the possibility that individuals with CSEM offending are heterogeneous in risk of reoffending.

The order of the offences in mixed CSEM offending ‒ whether a CSEM offence was committed before or after a contact sexual offence – and its association with risk factors and future reoffending has not yet been examined. Greater offence severity is generally unrelated to recidivism for individuals adjudicated for sexually motivated offences (Hanson & Bussière, 1998); however, severity predicts recidivism in other offending populations. For example, index offence severity predicted short-term recidivism (1 year) in a sample of adolescents adjudicated for criminal offences (e.g., Mulder et al., 2010), the severity of index offences predicts reoffending as well as the speed of reoffending in individuals adjudicated for interpersonal violence (Goldstein et al., 2016; Hilton & Eke, 2017; Hilton et al., 2004), and two large cohort studies of individuals incarcerated in federal institutions in Canada found that offence severity is predictive of readmission to Canadian federal custody (Offence Severity Record [OSR] of the Static Factor Assessment; Helmus & Forrester, 2017; Perley-Robertson et al., 2019).

## Current Study

The current study examined the extent to which the temporal order of sexual offences by men who have committed CSEM offences is risk-relevant. Using the first two sexual crimes in the criminal record, we expected to find three patterns^1^1Terminology is an important consideration. The current study uses terminology common to the gateway hypothesis literature for ease of understanding. It is not meant to devalue the severity of CSEM offending.: *de-escalation pattern*, that is, men whose first sexual offence was a contact sexual offence followed by a CSEM offence; *Stable pattern*, that is men who committed a CSEM offence and whose next sexual offence was a CSEM offence; and, (3) *escalation pattern*, that is men who committed a CSEM offence and whose next sexual offence was a contact sexual offence. Cases where someone committed an offence producing CSEM with a child were categorized as a contact offence. Exploratory analyses were conducted to examine if CSEM groups differed on risk-relevant factors, as well as 5- and 7-year recidivism rates (start of follow-up: opportunity after the second sexual offence). Given the aim of the study is to examine CSEM offending, we did not examine the stable contact offending (contact to contact) pattern.

## Method

### Participants

**Figure 1:**
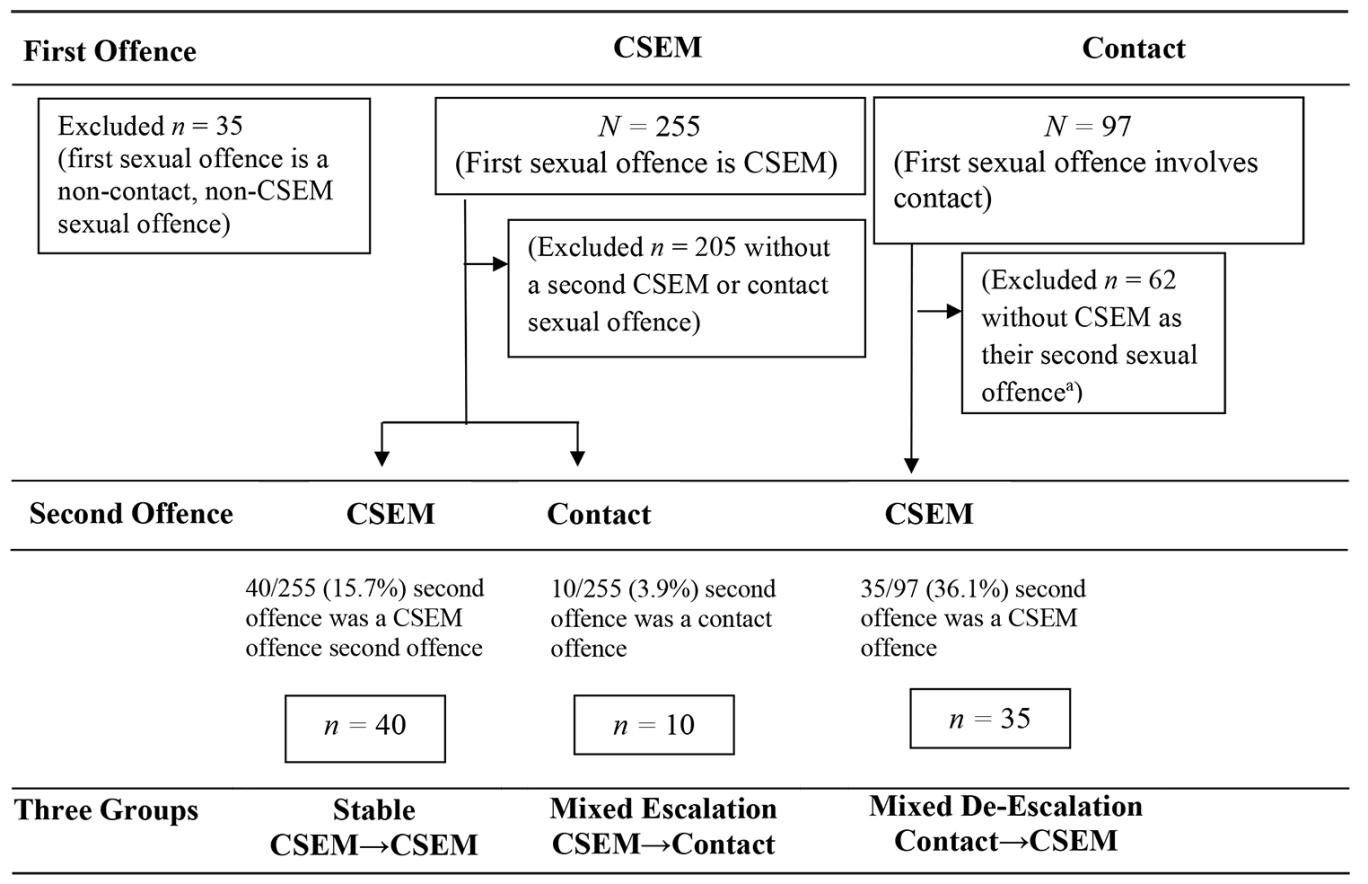
Group Selection Flow-Chart (N = 387) *Note.* CSEM = Child Sexual Exploitation Materials. ^a^All groups had to have at least one CSEM offence; those who did not have a CSEM offence in their first two sexual offences were excluded. Allegations were included in the groupings. Some individuals had both CSEM and contact offences at the same occasion; the most severe offence (contact) was used for the groupings.

The current study presents a re-analysis of a sample of 387 men adjudicated for CSEM offences prior to 2011 (i.e., possessing, distributing, accessing, or making CSEM) in a large Canadian province previously reported in Eke et al. (2019), but with an extended follow-up period until 2018 (also used in Babchishin et al., 2022; average follow-up 20 years, range from 9 to 61 years following their first sexual crime). There was no preselection of cases; all available closed CSEM case files that met the original project criteria were included if there was sufficient information for the current study. We defined the index offences as the first two sexual offences in their criminal record, to determine order patterns (see Figure 1). On average, the individuals were 39 years (*SD* = 13; range = 18 to 76) old at release from their first CSEM offence.

There were 255 individuals whose first ever sexual offence was a CSEM offence and 97 individuals whose first ever sexual offence was a contact sexual crime. There were also 35 individuals whose first crime was another type of sexual crime (e.g., non-contact sexual crime, excluding CSEM); these cases were excluded from the current study. Of those remaining individuals whose first sexual crime was a CSEM or a contact sexual crime (*n* = 352), two-thirds (*n* = 229) did not have a second CSEM or contact sexual crime during the follow-up period. Of the 123 with two sexual offending occasions, 38 individuals whose first crime was contact sexual offence subsequently committed a sexual contact offence and were removed from the dataset because this group (contact to contact sexual offences) was not relevant to the purpose of the current study.

Of the 85 remaining men with two sexual offending occasions, the most common (47%, *n* = 40) pattern was a *stable pattern* (committed a CSEM offence and reoffended with a CSEM offence as their second reoffence), 41% (*n* = 35) fit a *de-escalation pattern* (committed a contact sexual offence and subsequently committed a CSEM offence as their next sexual offence); and 12% (*n* = 10) fit an *escalation* pattern (i.e., had a CSEM offence and subsequently reoffended with a contact sexual offence).

### Procedure

The complete offending timeline and history of men with CSEM offending were available through to 2018. Offence history and offence details, demographic information, and psychological variables (available to a lesser extent) were coded from police file information in two previous studies (Eke et al., 2019; Seto & Eke, 2015). The criminal history spanned the criminal record, using police occurrence reports, the Canadian Police Information Centre (CPIC), and investigative file information from the collaborating police services in the original research. Charges and convictions were used to assign individuals into offending patterns, but we also used allegations (i.e., suspect or police investigation cases, but without resulting charge) to assign participants into groups when present (9% in the first offence [8/85], 8% in the second offence [7/85]). In cases where the sexual offence cluster included both contact and CSEM offending (e.g., producing CSEM offence with contact sexual victims), we characterized this offence as a contact offence (7/45 men with contact sexual offences; 16%). The original scoring of demographic variables and psychological variables were used, which had good interrater reliability in the original study (*r* = .94 to 1.00, kappa ranging from .70 to 1.00; Seto & Eke, 2015). The interrater reliability of the extended follow-up data was also good (ICC = .96 to 1.00 [*Mdn* = 1.00]; κ ranged from .50 to 1.00, *Mdn* = .93; Babchishin et al., 2022).

### Measures

The archival database was administrative and contained demographic information, criminal history, and some psychological variables. We sorted these into two main domains: items indicative of antisocial tendencies and items indicative of sexual interest in children. The archival dataset also included the Child Pornography Offender Risk Tool (CPORT, Seto & Eke, 2015): a 7-item structured tool to assess the likelihood of future sexual offending among men with CSEM offences. We rescored the CPORT to reflect the CPORT score at the time of the individual’s second sexual offence – the offence used to create the individual’s offending pattern. As with the method used to group individuals, information about allegations was available and we report CPORT scores with and without the inclusion of allegations of prior offending (allegations are not currently included in the formal scoring of the tool.^2^2see https://www.researchgate.net/project/Child-Pornography-Offender-Risk-Tool-CPORT


#### Antisocial Tendencies

Age at police involvement (i.e., age at first police involvement, age at first CSEM offence), unemployed at the first CSEM offence, prior criminal history (i.e., total number of offences, and total number of any violent offences *prior* to their first sexual offence; these criminal history variables included allegations), substance misuse items (the Violence Risk Appraisal Guide [VRAG; Harris et al., 1993] items: severe drug use problem, severe alcohol use problem, and driving under the influence charge scored at first CSEM offence).

#### Sexual Interest in Children

Never married at the time of the first CSEM offence, number of sexual crimes in their index clusters (total number of CSEM offences including making CSEM offences, total number of contact sexual offences, total number of contact sexual victims) were extracted from the dataset. CPORT items indicative of sexual interest (CPORT Item 5 Pedophilia or hebephilia, CPORT Item 6 More boy than girl CSEM, and CPORT Item 7 More boy nude and other images) were recoded to reflect the second CSEM offence, when applicable.

#### Recidivism

Recidivism following their second sexual offence was primarily based on official charges or convictions but also included allegations (i.e., suspect or police investigation cases, but without resulting charge). We examined six types of recidivism: (1) CSEM offence, (2) contact sexual offence, (3) any sexual offence – any offence that was considered sexually motivated, (4) non-sexual violent offences, (5) violent offence – all crimes that involved direct confrontation with the victim (including contact sexual offences, but excluding non-contact sexual offences and sexually motivated breaches), and (6) any offence (sexual, violent, non-violent). To allow for comparisons with the published literature, we computed 5-year recidivism rates. We also present 7-year follow-up results because we had sufficient data to do so.

### Data Analysis

Men adjudicated for CSEM offences were classified according to their offending patterns. We then compared these groups on indicators of antisocial tendencies and sexual interest in children. We privileged effect sizes for interpretation of these exploratory analyses. Cohen’s *d* was calculated using the formula from Cohen (1988) for continuous variables and estimated from dichotomous variables using the formula from Sánchez-Meca et al. (2003) and adding 0.5 to each cell to allow for *d* calculation with empty cells (Fleiss, 1994). Cohen’s *d*s of .20, .50, and .80 are considered small, moderate, and large effects (Rice & Harris, 2005). Next, we compared the recidivism rates of the groups. The sample sizes for the recidivism analyses are small, as we required at least five and seven years following their latest sexual offence used for grouping.

## Results

### Group Differences

The de-escalation group had the youngest age at first police involvement and more prior offending than the escalation group (absolute *d*s ranging from .12 to .31) and stable group (absolute *d*s ranging from .27 to .32; see Table 1). The escalation group had more alcohol use, as well as charges for driving under the influence than the de-escalation group (absolute *d*s ranging from 0.39 to 0.58) and the stable group (absolute *d*s ranging from .85 to 1.20). The stable group scored lowest on substance use issues and had the fewest number of prior offences among the three groups. The stable group was also the least likely to be unemployed.

**Table 1:** Characteristics of CSEM Recidivists as Function of Order of Offending

Variable	Mixed De-Escalation: Contact → CSEM	Mixed Escalation: CSEM → Contact	Stable: CSEM → CSEM	Comparisons		
				Mixed Escalation vs Mixed De-Escalation^a^	Mixed Escalation vs Stable^b^	Mixed De-Escalation vs Stable^c^
	*M* (*SD*) or *n/N* (%)			*d* [95% CI]		
Antisocial Tendencies						
Age at first police involvement	27.5 (9.8), *n* = 35	32.2 (15.5), *n* = 10	30.4 (11.4), *n* = 39	-0.42 [-1.12, 0.29]	-0.15 [-0.84, 0.55]	0.27 [-0.19, 0.73]
Age at first CSEM offence	38.4 (13.2), *n* = 35	38.0 (12.2), *n* = 9	33.1 (10.9), *n* = 40	0.03 [-0.70, 0.76]	-0.44 [-1.17, 0.29]	-0.44 [-0.90, 0.02]
Unemployed at the first CSEM offence	5/35 (14.3%)	1/10 (10.0%)	2/40 (5.0%)	-0.08 [-1.26, 1.10]	0.54 [-0.76, 1.84]	0.31 [-0.72, 1.33]
Prior number of non-sexual violent offences	0.46 (0.92), *n* = 35	0.20 (0.42), *n* = 10	0.23 (0.48), *n* = 40	-0.31 [-1.01, 0.40]	-0.05 [-0.75, 0.64]	0.32 [-0.13, 0.78]
Prior number of offences	2.20 (4.65), *n* = 35	1.70 (2.45), *n* = 10	1.20 (2.49), *n* = 40	-0.12 [-0.82, 0.59]	0.20 [-0.49, 0.90]	0.27 [-0.18, 0.73]
CPORT Total Score	3.26 (1.26), *n* = 34	–^d^	3.19 (1.31), *n* = 32	–	–	0.06 [-0.42, 0.54]
CPORT Total Score with the use of allegations	3.51 (1.15), *n* = 35	3.40 (0.84), *n* = 10	3.05 (1.36), *n* = 40	-1.10 [-0.80, 0.60]	0.27 [-0.42, 0.97]	0.37 [-0.09, 0.82]
Severe drug use problem (VRAG item)	0/15 (0.0%)	0/5 (0.0%)	0/23 (0.0%)	–	–	–
Severe alcohol use problem (VRAG item)	2/19 (10.5%)	1/6 (16.7%)	1/23 (4.3%)	0.39 [-0.97, 1.75]	0.85 [-0.63, 2.34]	0.46 [-0.82, 1.75]
Driving under the influence (DUI)	5/35 (14.3%)	3/10 (30.0%)	2/40 (5.0%)	0.58 [-0.37, 1.52]	**1.20 [0.10, 2.29]**	0.62 [-0.33, 1.57]
Indicative of Interest in Children						
Total number of CSEM offences at index	1.17 (0.38), *n* = 35	1.10 (0.32), *n* = 10	2.00 (0.00), *n* = 40	-0.19 [-0.90, 0.51]	**-6.58 [-8.04, -5.11]**	**-3.18 [-3.86, -2.49]**
Making CSEM offences at index	0.37 (0.60), *n* = 35	0.20 (0.42), *n* = 10	0.58 (0.68), *n* = 40	-0.30 [-1.01. 0.40]	-0.34 [-1.03, 0.36]	-0.32 [-0.77, 0.14]
Never married at the first CSEM offence	16/33 (48.5%)	3/10 (30.0%)	23/38 (60.5%)	-0.43 [-1.30, 0.44]	-0.71 [-1.58, 0.15]	-0.29 [-0.85, 0.28]
Volunteered with kids	3/34 (8.8%)	0/10 (0.0%)	4/37 (10.8%)	-0.69 [-2.51, 1.34]	-0.63 [-2.45, 1.19]	-0.12 [-1.01, 0.78]
CPORT Item 5 (pedophilia or hebephilia)	15/32 (46.9%)	6/8 (75.0%)	31/34 (91.2%)	0.65 [-0.32, 1.63]	-0.75[-1.86, 0.36]	**-1.41 [-2.19, -0.62]**
CPORT Item 6 (more boy than girl CSEM)	3/33 (9.1%)	0/10 (0.0%)	10/37 (27.0%)	-0.53 [-2.38, 1.31]	-1.26 [-3.03, 0.51]	-0.73 [-1.53, 0.07]
CPORT Item 7 (more boy nude and other images)	4/31 (12.9%)	0/10 (0.0%)	10/37 (27.0%)	-0.74 [-2.57, 1.07]	-1.26 [-3.03, 0.51]	-0.51 [-1.26, 0.23]
Number of minor victims in contact sexual offence	0.77 (0.43), *n* = 35	0.60 (0.56), *n* = 10	0.00 (0.00), *n* = 40	-0.38 [-1.09, 0.32]	**2.68 [1.81, 3.55]**	**2.65 [2.03, 3.27]**

*Note.* Bolded values represent a statistically significant group difference *p* < .05.

^a^Mixed escalation group was the reference group; positive *ds* indicate that mixed escalation group had more risk-relevant problems than the mixed de-escalation group. ^b^Mixed escalation group was the reference group; positive *ds* indicate that mixed escalation group had more risk-relevant problems than the stable group. ^c^Mixed de-escalation group was the reference group; positive *ds* indicate that mixed de-escalation group had more risk-relevant problems than the stable group. ^d^Only one participant scoring a 4 was in this cell; thus, *ds* for CPORT total score that included this group (i.e., mixed escalation) was not computed.

Indicators of sexual interest in children were based on criminal history, on characteristics of their CSEM collection (Items 6 and 7 of the CPORT), and on their self-reported or prior diagnosis of sexual interest in children (Item 5 of the CPORT). Unsurprisingly, both the escalation and de-escalation groups had more contact sexual offending victims than the stable group, and the stable group had more CSEM offences than the two mixed offending groups given our grouping definition. The stable group had more making CSEM offences than the de-escalation (absolute *d* = 0.32) and escalation groups (absolute *d* = 0.34). The Canadian legal definition for ‘making’ CSEM is broader than the legal definition of ‘production’ in the United States and included morphing images, reproducing images (e.g., saving images to CDs, printing text stories), writing text stories about sex with children, or creating websites for sharing CSEM. None of the making charges for the stable group included documentation of abuse.

The stable group had more indicators of sexual interest in children based on their CSEM collections (more boy than girl images), reported sexual interest in children (or had a prior diagnosis for pedophilic disorder or hebephilic disorder as Paraphilic Disorder Not Otherwise Specified), and volunteered with children more than both mixed groups (absolute *d*s ranging from .12 to 1.41). The stable group, however, was more likely to be married (60.5%) than both the de-escalation and escalation groups (48.5% and 30%, respectively).

### Recidivism

We examined 5-year and 7-year recidivism rates, following release from the latest sexual offence in the index offending cluster (see Table 2). The escalation group had the highest 5- and 7-year observed recidivism rates of contact sexual recidivism (14.3% for both follow-up times) compared to the de-escalation group (6.5% at 5-year, 12.9% at 7 years) and stable group (8.3% for both follow-up times). The escalation group also had the highest any crime recidivism (57.1% at both follow-up times) and non-sexual violent recidivism rates (28.6% at both follow-up times) but showed the lowest recidivism rates of CSEM offences (14.3%).

**Table 2:** Observed 5-Year and 7-Year Recidivism Rates of Three CSEM Recidivist Groups

Groups	Any crime (sexual, violent, non-violent)		Non-sexual Violent		Violent (including contact sexual)		Any Sexual		Contact Sexual		CSEM	
	5-year	7-year	5-year	7-year	5-year	7-year	5-year	7-year	5-year	7-year	5-year	7-year
De-Escalation: (Contact → CSEM)	9/31 (29.0%)	11/31 (35.5%)	2/31 (6.5%)	3/31 (9.7%)	3/31 (9.7%)	6/31 (19.4%)	9/31 (29.0%)	11/31 (35.5%)	2/31 (6.5%)	4/31 (12.9%)	7/31 (22.6%)	7/31 (22.6%)
Escalation: (CSEM → Contact)	4/7 (57.1%)	4/7 (57.1%)	2/7 (28.6%)	2/7 (28.6%)	3/7 (42.9%)	3/7 (42.9%)	2/7 (28.6%)	2/7 (28.6%)	1/7 (14.3%)	1/7 (14.3%)	1/7 (14.3%)	1/7 (14.3%)
Stable: (CSEM → CSEM)	8/24 (33.3%)	9/24 (37.5%)	0/24 (0.0%)	0/24 (0.0%)	2/24 (8.3%)	2/24 (8.3%)	8/24 (33.3%)	9/24 (37.5%)	2/24 (8.3%)	2/24 (8.3%)	6/24 (25.0%)	8/24 (33.3%)

*Note.* Four men with de-escalation (Contact → CSEM) CSEM offending, 3 with Escalation (CSEM → contact) CSEM offending, and 16 with stable (CSEM → CSEM) offending did not have a 7-year follow-up time following their latest sexual offence. Such cases, thus, were excluded when calculating the recidivism rates. Recidivism events were primarily based on charges and convictions but also included police allegations.

**Figure 2:**
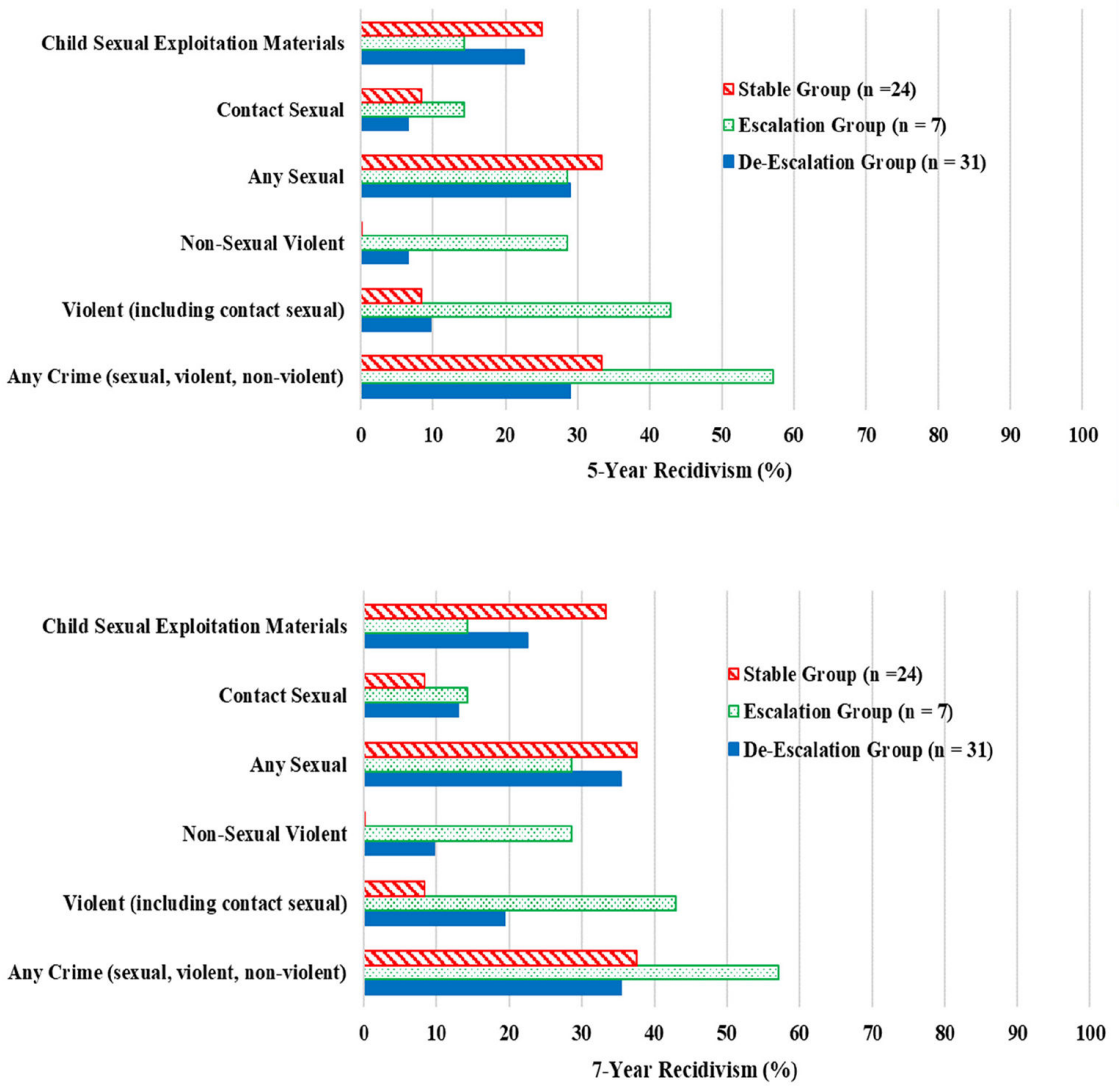
Observed 5- (Upper) and 7-Year (Lower) Recidivism Rates *Note*. For the 5-year follow-up, recidivism event counts that were based on allegations and not official charges or convictions were: one violent offence allegation, two contact sexual offence allegations, and one CSEM offence allegation. For the 7-year follow-up, there were two violent allegations, three contact sexual allegations, and one CSEM allegation. There were no allegations for non-sexual violent offences in our sample.

The CSEM recidivism rates were the highest for the stable group (25% at 5-year, 33% at 7-year), followed by de-escalation group (23% for both 5-year and 7-year; Figure 2).

## Discussion

Our study adds to the literature that finds that CSEM offending is not a gateway to contact sexual offending for most men with CSEM offences. After an average 20-year follow-up from first sexual crime (follow-up range of 9 to 61 years), two-thirds of men with CSEM offences were not detected for a reoffence with a contact sexual or CSEM offence. Of the 85 men with at least two sexual crimes during this time period, 5 in 10 fit the stable pattern, 4 in 10 fit the de-escalation pattern, and 1 in 10 fit the escalation pattern.

We conducted analyses to identify risk profiles and recidivism rates (5- and 7-years, following the opportunity after the second occasion for a sexual crime) of the three groups. Using a representative cohort of men with CSEM offending, we found that men who escalate with a new contact sexual offence after a CSEM offence tend to score higher on indicators of substance use than those who reoffend with a CSEM offence. We found that the de-escalation group had an earlier age of onset than the two other groups, had more prior criminal history, and fewer indicators of sexual interest. The stable group were the least likely to be unemployed and more likely to be married (despite scoring higher on sexual interest in children and having greater indicators of sexual interest in children than the other groups). Employment and marriage have been found to be protective against sexual offending (Laws & Ward, 2011). The current study suggests that these factors protect against contact sexual offending, but may not protect against CSEM offending, because 33% of the stable group reoffended with another CSEM offence after seven years.

Mixed sexual offending men have higher recidivism rates than men with CSEM-exclusive offending pattern (e.g., Elliott et al., 2019). The current study found that among mixed offending men, men who fit the escalation pattern are at a greater risk to reoffend with any crime, non-sexual violent offences, violent offences, and contact sexual offences than men who fit the de-escalation pattern, as well as those who fit the stable pattern. As a function of our methodology – all individuals had to have at least two sexual offences ‒ observed 5- and 7-year recidivism rates were quite high for all three groups; indeed, the stable group had a higher 5-year contact sexual recidivism rate (8%) than typical samples of individuals with CSEM exclusive offending (typically 0-2%; see Babchishin et al., 2018, for review). In other words, having more than one sentencing occasion for a CSEM offence is a risk factor for future contact sexual offending, as well as further CSEM offences.

In sum, the temporal order of sexual offending is risk-relevant for CSEM offending. Men with CSEM offences who then committed a contact sexual offence (i.e., escalation pattern) were at a higher risk to reoffend than those with a stable pattern (CSEM to CSEM offending) and de-escalation pattern (contact to CSEM offending). Previous research has identified two distinct groups of individuals with CSEM offences: CSEM-exclusive (sexual offences are exclusively CSEM offences) and mixed CSEM offending (individuals with contact sexual offences and CSEM offences). The current study suggests that the number and ordering of sexual offences is risk-relevant for CSEM offending. Mixed CSEM offending men are heterogeneous in their risk profile and their risk to reoffend and some of this heterogeneity can be explained by the number and order of sexual offences in their criminal history.

### Practical Implications

Correctional programs that tailor intervention intensity to the risk profiles of participants are more effective at reducing reoffending (Andrews & Bonta, 2010; Bonta & Andrews, 2016). The current study found that men with CSEM offending, including mixed CSEM offending, vary on risk for recidivism; one size does not fit all individuals with CSEM offending. First, men with CSEM offending and an additional sexual crime (whether contact or CSEM) have recidivism rates similar to or higher than men with typical contact sexual offending, necessitating greater treatment intensity than men with a single CSEM offence. Second, among mixed offending individuals, those who fit the escalation pattern are at higher risk to reoffend than those who fit the de-escalation pattern. Treatment programs that allow for varying intensity of treatment among individuals with CSEM offending may provide better recidivism reduction than programs that treat all individuals with CSEM offending similarly. Such programs have yet to find a statistically significant reduction in recidivism (Beier et al., 2015; Wild et al., 2020; also see Table A.8 in Mews et al., 2017). Risk tools can be used to help sort individuals with CSEM offending in terms of their estimated risk to reoffend (for review, see Brown, 2022).

Among those with at least two sexual offending occasions, most fit the stable or de-escalation pattern, with 1 in 10 fitting the escalation pattern. The escalation group had more indicators of substance use and had higher rates of recidivism for all but CSEM reoffending. As such, men with CSEM offences who also have substance use problems may be particularly at risk to commit a new contact sexual offence. However, additional prospective research studies that include substance use measures are needed to replicate this finding.

Nonsexual recidivism rates, whether violent or nonviolent, were notable for all groups. As such, general recidivism tools (in addition to those designed to predict sexual recidivism) could be useful for individuals with CSEM offending. For a proportion of individuals with CSEM offending, treatment should include interventions that target general criminality. Men with mixed CSEM offending may particularly benefit from general interventions. In contrast, the stable group may benefit most from interventions that target and help manage their atypical sexual interests. All groups likely would benefit from potentially facilitating factors associated with CSEM use (e.g., coping, use of leisure time; Seto, 2013). Given the heterogeneity in CSEM offending observed in the current study, general criminality (e.g., antisocial personality traits, antisocial friend) and sexual criminality (e.g., atypical sexual interests, sexual preoccupation, sexualized coping; Mann et al., 2010) assessments would be valuable in directing interventions (e.g., supervision, programming).

### Limitations and Future Directions

A large sample size and lengthy follow-up are required to investigate the offending pattern of men with CSEM offending. Despite a follow-up period from the first recorded sexual offence ranging from 9 to 61 years (*M* = 20 years), two-third of our sample of men with CSEM offending were not detected for a new contact sexual or CSEM offence. The analyses were based on the one-third that did commit a reported second sexual offence during the follow-up period (*n* = 85), and most of these were classified in the stable and de-escalation groups. As a result, our analyses (especially recidivism analyses) were based on a small sample size. The small sample size precluded us from examining the predictive accuracy of the CPORT for these subgroups. Future research aiming to examine the predictive accuracy of risk tools for mixed CSEM subgroups would likely need to combine datasets because most men with CSEM offending are expected to exclusively have one detected CSEM offence or fit the stable group (two CSEM offences).

We also supplemented official charges and conviction with allegations to sort individuals into offending groups. This meant that 8 individuals (7 contact to CSEM and 1 CSEM to CSEM) were sorted based solely on allegations, without charges or convictions. Reasons for allegations to be closed without charges were lack of supporting or reliable evidence, the suspect’s identity could not be confirmed, or the case was closed as “unsolved”. Many of these allegations were older, from the 1980s or early 1990s, and involves cases where the full police reports were no longer available. Although we used allegations to inform group allocation for only a minority of the sample (8 of 85), it is possible that the use of allegations could have reduced group differences to the extent that the allegations were incorrect and resulted in misclassification. We also included individuals with mixed offending in the contact group. Namely, if someone had both CSEM and contact sexual offences in the same sentencing occassion, we classified this individual in the contact sexual offence group. Future research, with larger sample sizes, may be able to examine these dual offending occassions (contact and CSEM in the same index) as a separate group. It is possible that men that follow a mixed stable pattern (i.e., contact and CSEM offence followed by another contact and CSEM offence) are particularly risky.

The current study drew criminal information from official criminal records, which is not a complete representation of all offences committed by a person. In addition, not all charges or convictions are included or submitted to the national system used in the current study, including some lesser or “summary” offences, offences diverted from the criminal justice system, and offences committed outside of Canada. Some charges or convictions can be purged, including offences committed as a youth or offences as an adult that were pardoned. Juvenile offending is therefore expected to be underrepresented in our sample, in part because the original sampling strategy necessitated that each individual had an adult conviction for CSEM. Also, youth offending data were known if noted in the file information, rather than it being based on an examination of juvenile records, which are sealed or purged if an individual remains offence-free as an adult for a number of years, as specified in the Canadian *Youth Criminal Justice Act*. For those individuals in our sample who were younger at the time of their index CSEM offence, there is perhaps a greater likelihood that juvenile data would be noted in an investigative file. Overall then, age at first police involvement may have been missing some juvenile offences and may be better described as age of first police involvement in adulthood. Studies have found that earlier age of onset is related to more extensive criminal trajectory (e.g., Le Blanc & Loeber, 1998; Moffitt & Caspi, 2003; Piquero & Moffitt, 2008). Common criticisms of age of onset studies include mainly methodological concerns such as the threshold between early and late onset being sample specific and inconsistent across studies and offence type being ignored despite studies finding that the first offence type is meaningful (Tzoumakis et al., 2013). In the current study, the de-escalation group were about 5 years younger than the escalation group and three years younger than the stable group. This finding is limited as our study did not have a complete coverage of criminal offences committed during adolescents for our sample.

One potential explanation for our patterns of findings is that the three groups may not be meaningfully different and instead are being examined at different stages in their criminal trajectory. Specifically, the current study used a cohort of men varying in age. Although we could look at criminal history, we only looked forward until 2018. It is possible that some men with a single sexual offence may have gone on to commit a new sexual offence should we have more complete information. Indeed, the stable group were five years younger than the escalation and de-escalation group at their first CSEM offences. In addition, although some escalation men committed new contact sexual offences, others committed new CSEM offences or committed no offence after 5- to 7-years follow-up. In other words, classification may change with time. Given that samples of individuals with CSEM offending are reaching longer follow-up times (e.g., > 20 years), more nuanced pictures of reoffending and stability of offending can finally be elucidated. Future research on CSEM offending should code the ordering of CSEM offending so that they can contribute to this line of research. The current study had a long follow-up from first offence (average of 20 years) and therefore could go beyond their initial sexual recidivism events. The sample size, however, was small and precludes strong conclusions, especially for recidivism rates.

The current study used a dataset based on available police reports and, as a result, the majority of our variables were criminal history and demographic indicators. Psychologically meaningful measures would contribute greatly to our understanding of the etiology and course of CSEM offending. In particular, it would be interesting to see how cognitions specific to CSEM offending and situational factors (e.g., alcohol use during the offence) may predict reoffending among individuals with CSEM offending (for review, see Steel et al., 2020). Mixed offending may be a promising indicator of antisociality as the current study found that both mixed groups (de-escalation and escalation) were higher on our indicators of antisociality than the stable group. Our antisociality indicators (substance use, criminal history), however, were quite limited. Future research would benefit from additional indicators of antisocial tendencies and other risk-relevant propensities. Such studies would be beneficial to direct treatment and management efforts of individuals adjudicated with CSEM offences. We also did not collect information on self-reported offending, and systematic reviews suggest a larger proportion of CSEM have contact sexual offences if based on self-report rather than official records (Seto et al., 2011). We were able to include allegations (i.e., suspect cases and allegation that resulted in police investigations), as well as arrests, charges, or convictions for crimes in our definitions of recidivism.

### Conclusion

The current study took advantage of the longer follow-up now available for CSEM studies to explore if the ordering of contact sexual and CSEM offending is risk-relevant among men with CSEM offending. We found that the ordering of CSEM offences is risk-relevant; mixed offending men are not all the same. Using the first two sexual crimes in their criminal trajectory to define groups, we found that escalation, de-escalation, and stable groups differ on risk-relevant indicators and 5- and 7-year recidivism rates. The escalation group had greater substance use issues, the de-escalation group had a younger age of onset and more prior offending, and the stable group had more indicators of sexual interest in children. We also found that a significant proportion (8%) of men who fit the stable pattern will go on to commit a contact sexual offence after 5- and 7-years. Management and treatment of individuals with CSEM offending should therefore be sensitive to their sexual offending patterns.

## Data Availability

Data are not publicly available due to existing data agreements.
